# Identification of aberrant circulating miRNAs in Parkinson's disease plasma samples

**DOI:** 10.1002/brb3.941

**Published:** 2018-02-19

**Authors:** Lei Chen, Junxiu Yang, Jinhui Lü, Shanshan Cao, Qian Zhao, Zuoren Yu

**Affiliations:** ^1^ Department of Neurology Tianjin Huan Hu Hospital Jinnan District, Tianjin China; ^2^ Tianjin Key Laboratory of Cerebrovascular and Neurodegenerative Diseases Tianjin China; ^3^ Department of Neurology Hospital of Integrated Traditional and Western Medicine Cangzhou China; ^4^ Research Center for Translational Medicine East Hospital Tongji University School of Medicine Shanghai China

**Keywords:** circulating miRNAs, diagnosis, Parkinson's disease

## Abstract

**Objective:**

To detect the aberrant expression of circulating miRNAs and explore the potential early diagnostic biomarkers in patients with Parkinson's disease (PD).

**Methods:**

Plasma samples were collected from 25 treatment‐naïve PD‐diagnosed patients and 25 healthy controls followed by a real‐time PCR‐based miRNA screening analysis of neuron disease‐related miRNAs.

**Results:**

A subset of miRNAs with aberrant expression levels in the plasma of PD‐diagnosed patients were identified including upregulation of miR‐27a and downregulation of let‐7a, let‐7f, miR‐142‐3p, and miR‐222 with the AUC values more than 0.8 derived from the receiver operating characteristic curves.

**Conclusions:**

The high sensitivity and specificity of the circulating miRNAs may enable early diagnosis of PD. The study provides a group of novel miRNA candidates for detecting PD.

## 1 INTRODUCTION

Parkinson's disease (PD) is the second most prevalent neurodegenerative disorder after Alzheimer's disease (AD) in the world. The PD prevalence is estimated around 1.7% in people over 65 years of age in China (Zhang et al., 2005). Parkinson's disease (PD) is characterized by the cardinal motor symptoms, such as bradykinesias, rigidity, postural instability, resting tremor, and nonmotor symptoms including psychiatric problems, autonomic disturbances, pain, fatigue and impaired cognition in executive functioning, memory and spatial behavior during the early stage of the disease (Chaudhuri & Odin, 2010; Vingerhoets, Verleden, Santens, Miatton, & De Reuck, 2003). The neuropathological hallmarks of PD include the loss of dopaminergic neurons in substantia nigra (SN) and the formation of neuronal protein inclusions known as Lewy bodies (LB) (Braak et al., 2003). Currently, all the therapeutics for PD only reduce the symptoms. There is no disease‐modifying way to treat PD when the full‐blown syndrome occurs. As such, it is important to identify useful biomarkers for early diagnosis of PD, especially before the onset of motor symptoms. The application of prodromal premotor treatment to PD is expected to slow down or stop the neurodegenerative process, leading to better quality of life of PD‐diagnosed patients.

miRNA is a class of small noncoding RNA with approximately 18–22 nucleotides in length. miRNAs mostly repress translation or induce mRNA degradation of the target genes by complementary interactions (Bartel, 2009; Griffiths‐Jones, Grocock, van Dongen, Bateman, & Enright, 2006). Around 1%–4% genes in the human genome encode for miRNAs and about one‐third of mRNAs are regulated by miRNAs (Lu et al., 2008). It has been well demonstrated that miRNAs play critical roles in many biological processes including cell fate determination, embryonic development, cell proliferation, differentiation, and apoptosis (Esquela‐Kerscher & Slack, 2006).

In the past years, miRNAs were found to be present in body fluids including blood, plasma, serum, saliva, urine, and milk (Mitchell et al., 2008). The extracellular miRNAs circulate in the blood of both healthy and diseased people, which are referred to circulating miRNAs. Circulating miRNAs may serve as novel diagnostic and prognostic biomarkers for human diseases. miRNA expression profiles in PD‐diagnosed patients have been analyzed and reported (Cardo et al., 2014; Hoss, Labadorf, Beach, Latourelle, & Myers, 2016), and the function of miRNAs in regulating PD has been thoroughly discussed (Filatova, Alieva, Shadrina, & Slominsky, 2012). Although there were a few studies examining the expression pattern of circulating miRNAs in the blood samples of PD‐diagnosed patients (Margis, Margis, & Rieder, 2011; Mushtaq et al., 2016), it is still unidentified for the PD‐specific miRNAs that can be applied for clinical diagnosis and/or prognosis of PD.

Here, we collected plasma samples from 25 PD‐diagnosed patients and 25 age‐matched healthy controls followed by a real‐time PCR‐based circulating miRNA analysis. A subset of miRNAs was identified to have aberrant levels in plasma samples of PD‐diagnosed patients.

## 2 MATERIALS & METHODS

### 2.1 Subject collection

This study was approved by the Ethics Committee of Tianjin Union Medical Center. All subjects were signed with the informed consent before entering into the study. We recruited participants from outpatients presenting at the movement disorder clinic of Tianjin Union Medical Center (Tianjin, China). Totally we recruited 25 patients who met PD diagnostic criteria according to the United Kingdom Parkinson's Disease Society Brain Bank Clinical Diagnostic Criteria (Hughes, Daniel, Kilford, & Lees, 1992), with 64.96 ± 8.66 years old for average age and 16 males/nine females for gender. The blood samples were collected before medication treatment. All patients were newly diagnosed and treatment‐naïve. The exclusion criteria include one of these: (i) presence of other neurological disease; (ii) other parkinsonian disorders such as drug‐induced parkinsonism, vascular parkinsonism, and/or atypical forms of parkinsonism; (iii) presence of psychiatric disorder; and (iv) unable to sign in the informed consent. Meanwhile, 25 healthy controls unrelated with the enrolled patients and matched by sex and age (range ±5 years) were enrolled from the Medical Examination Clinic of Tianjin Union Medical Center.

### 2.2 Plasma collection

Five millilitre of peripheral blood sample from each subject was collected in an EDTA‐treated tube, followed by immediate centrifugation at 1,000 × *g* for 5 min at 4°C. The supernatant plasma was aliquoted to 200 μl/tube and stored at −80°C for further analysis.

### 2.3 Total RNA extraction from plasma

TRIzol reagent (Life Technologies) was used for total RNA extraction from plasma samples. Briefly, 200 μl plasma was mixed well with 1 ml TRIzol reagent to denature proteins at room temperature for 10 min. Then, 200 μl chloroform was added and mixed thoroughly followed by incubation on ice for 5 min until showing two layers. After centrifuging at 12,000 × *g* for 15 min at 4°C, the clear upper layer liquid was transferred into a new tube without touching the low layer, and mixed with same volume of isopropanol. After incubation at −20°C overnight, the mixture was centrifuged at 12,000 × g for 15 min at 4°C to precipitate RNA. Then discarding the supernatant carefully, and washing the RNA pellet using 75% ethanol followed by a centrifugation at 12,000 × *g* for 10 min at 4°C, followed by removing the ethanol, air drying the pellet at room temperature, and then resolving the total RNA in 10 μl of DNase/RNase‐free H2O. 1 μl RNA solution was applied on NanoDrop (Thermo Scientific, USA) for RNA concentration and quality analysis. Only RNAs with ratios of both 260 nm/280 nm and 260 nm/230 nm no <1.80 were used for further analysis. The miRNA quality and quantity were further confirmed using the Agilent 2100 bioanalyzer.

### 2.4 Synthesis of the first‐strand cDNA for miRNA

A commercially available kit, M&G miRNA Reverse Transcription kit (miRGenes, Shanghai, China) was used to prepare the first‐strand cDNA of plasma miRNAs following the manufacturer's instruction. 200 ng of purified total RNA was used for miRNA reverse transcription. The synthesized cDNA was diluted by 1:500 for real‐time PCR analysis.

### 2.5 miRNA expression profiling analysis

Around 2588 mature miRNA molecules have been identified or predicted from human samples (miRBase Release 21). In order to identify the PD‐specific or PD‐related miRNAs accurately and effectively, a neurodegenerative disease‐related miRNA panel which covers 91 neuron disease‐related miRNAs (being selected according to the literature) (Asikainen et al., 2010; Cardo et al., 2013; Filatova et al., 2012; Junn et al., 2009; Khoo et al., 2012; Mouradian, 2012; Salta & De Strooper, 2012; Serafin et al., 2014; Shioya et al., 2010; Wang et al., 2008), two reference small RNAs and one NTC (no template control) were customized and prepared by miRGenes. A list of the miRNAs in the panel is shown Table 1. In addition, two wells for miR‐16 (locations A3 and H10) on each panel were designed for testing the reproducibility. The cDNA was prepared as described above. The sequences of forward primers for the tested miRNAs are available upon request. All primers were tested for specificity and efficiency to amplify miRNAs. NTC was used for negative control of the PCR analysis. Universal reverse primer was provided by miRGenes. The SYBR Green Master Mix was purchased from ABI (Applied Biosystem, Life Technologies, USA). The ABI 7500 Sequence Detection System was used for quantitative real‐time PCR analysis. 5s rRNA was used for normalization. The results were analyzed by Mev (version 4.9) and MedCalc 15.2.2 software.

**Table 1:** Customized neuron disease‐related miRNA panel

	1	2	3	4	5	6	7	8	9	10	11	12
A	H2O (NTC)	5s rRNA	miR‐16	let‐7a	let‐7b	let‐7c	let‐7d	let‐7e	let‐7f	let‐7 g	let‐7i	miR‐1
B	miR‐10b	miR‐100	miR‐103a	miR‐106a	miR‐106b	miR‐107	miR‐125a	miR‐125b	miR‐126	miR‐127‐3p	miR‐128a	miR‐130a
C	miR‐130b	miR‐132	miR‐133b	miR‐139	miR‐141	miR‐142‐3p	miR‐143	miR‐144	miR‐145	miR‐150	miR‐151‐5p	miR‐155
D	miR‐15a	miR‐15b	miR‐17	miR‐181a	miR‐181b	miR‐185	miR‐19a	miR‐19b	miR‐191	miR‐200a	miR‐200b	miR‐200c
E	miR‐204	miR‐21	miR‐221	miR‐222	miR‐23a	miR‐24	miR‐26a	miR‐27a	miR‐27b	miR‐29a	miR‐29b	miR‐30a‐5p
F	miR‐30c	miR‐30d	miR‐30e‐5p	miR‐320a	miR‐320b	miR‐323a	miR‐326	miR‐34a	miR‐34c	miR‐370	miR‐382	miR‐383
G	miR‐409‐3p	miR‐422a	miR‐423‐3p	miR‐423‐5p	miR‐433	miR‐484	miR‐485	miR‐485*	miR‐486	miR‐487b	miR‐491	miR‐539
H	miR‐638	miR‐665	miR‐762	miR‐770	miR‐874	miR‐92a	miR‐93	miR‐935	miR‐99b	miR‐16	5srRNA	U6

### 2.6 Data analysis

The miRNAs with crossing threshold (Ct) value over 35 were considered as undetectable. For each panel, miRNAs were normalized by 5s rRNA. The relative expression level of miRNAs was calculated using the 2^−△△Ct^ method.

The Mann–Whitney test was used for statistical analysis using IBM SPSS Statistics 21.0 software. A Bonferroni correction was applied to account for multiple comparisons of the miRNAs in the panel. Adjusted *p*‐value ≤.05 was settled as the limit of statistical significance. Only those miRNAs with fold change ≥2 and adjusted *p*‐value ≤.05 were considered as significantly different.

To examine the prediction capability, receiver operating characteristic (ROC) curves were generated on those miRNAs that were identified to be significant by the Mann–Whitney test. The ROC analyses were performed by MedCalc Software. The miRNAs with AUC value more than 0.8 were considered having high sensitivity and specificity.

## 3 RESULTS

### 3.1 Preparation of neuron disease‐related miRNA panel

A customized PD‐miRNA panel was designed and prepared as described in methods. Although it has been in argument and discussion on reference strategies for normalizing circulating miRNAs, there is still no consensus (Ma et al., 2012). 5s rRNA, U6 snRNA, and miR‐16 are frequently used as internal normalization controls (Luo et al., 2009; Pineles et al., 2007; Zhao et al., 2010). In this study, U6 snRNA showed low abundance in the plasma samples. The miR‐16 levels showed big variation. In comparison with U6 snRNA and miR‐16, 5s rRNA had more stable levels in all the plasma samples with Ct values 20~25. As such 5s rRNA was selected as reference for normalization of miRNAs.

### 3.2 Real‐time PCR analysis of the miRNAs

**Figure 1:**
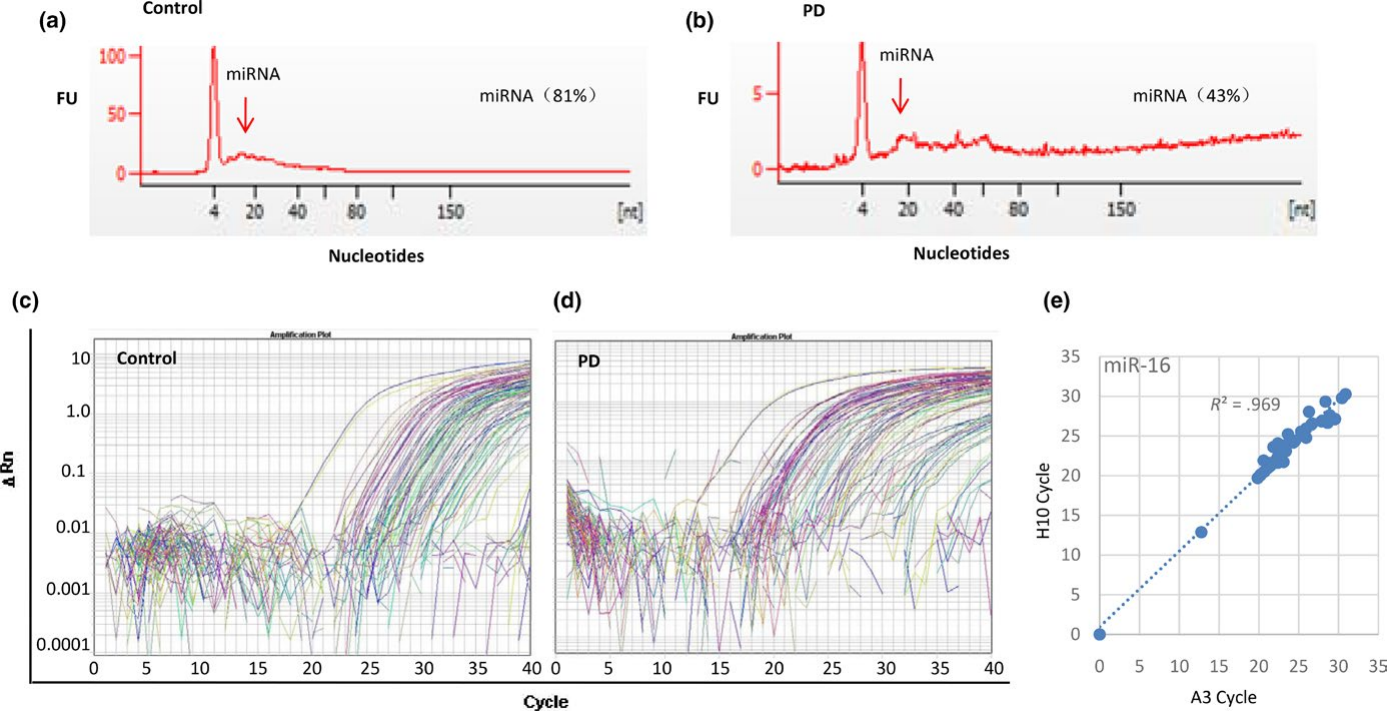
Real‐time PCR analyses of plasma samples from PD‐diagnosed patients and normal controls using the PD‐miRNA panel. (a) The RNA analysis using the Agilent 2100 bioanalyzer of the representative control sample. (b) The RNA analysis using the Agilent 2100 bioanalyzer of the representative Parkinson's disease (PD) sample. (c) The amplification curves of real‐time PCR analysis on miRNAs in the representative control sample. (d) The amplification curves of real‐time PCR on miRNAs in the representative PD sample. (e) Scatter plot showing the Ct (cycle threshold) values of miR‐16 at location A3 in the panel was perfectly repeated by that at location H10 in all 50 samples, forming a very good linear correlation with *R* ^2^ = .9697, demonstrating the reproducibility of the miRNA analysis by real‐time PCR

In order to identify the miRNAs that were differentially expressed in the plasma of the PD‐diagnosed patients, plasma samples from 25 PD‐diagnosed patients and 25 disease‐free controls were collected, total RNAs were isolated from the plasma samples, and first‐strand cDNA for miRNAs were synthesized following the protocols as described in M&M section. Real‐time PCR analyses were performed using the customized PD‐miRNA panels and cDNAs from 25 PD‐diagnosed patients and 25 controls. The quality and quantity of the miRNA population in total RNA in the plasma samples were confirmed using the Agilent 2100 bioanalyzer (Figures S1and S2). Figure 1a,b showed the results of the representative normal control and PD samples. The miRNAs were well amplified from the cDNAs by PCR analyses as shown in Figure 1c for the representative control sample and Figure 1d for the representative PD sample. The reproducibility of the miRNA analyses was demonstrated by the miR‐16 duplicates (locations A3 and H10) on each panel. As sown in Figure 1e, the Ct (cycle threshold) values for miR‐16 at A3 were perfectly repeated by that at H10 in all 50 samples, forming a good linear correlation with *R* ^2^ = .9697.

### 3.3 miRNA expression profiling in the plasma of PD‐diagnosed patients and control

**Figure 2:**
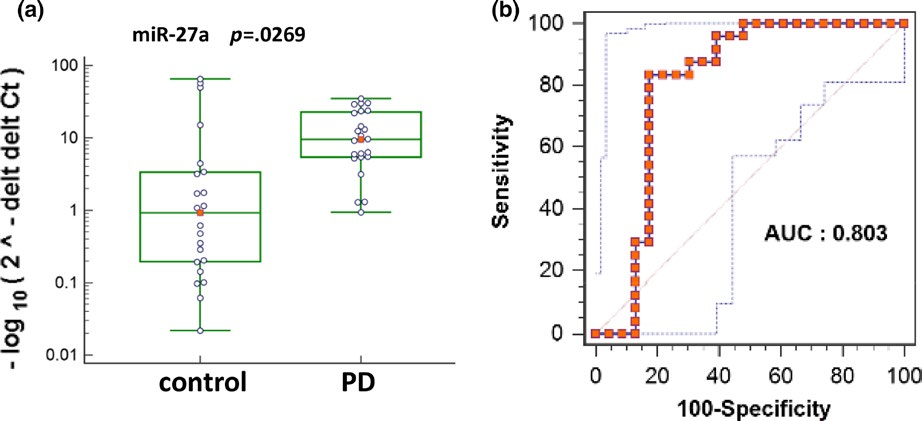
miR‐27a showed upregulation in the plasma of PD‐diagnosed patients compared with normal controls. (a) Scatter plot showing the expression levels of circulating miR‐27a in Parkinson's disease (PD) and control samples. *p* = .0269. (b) Receiver operating characteristic curves (ROC) of miR‐27a were generated using MedCalc Software, with the AUC value 0.803

**Figure 3:**
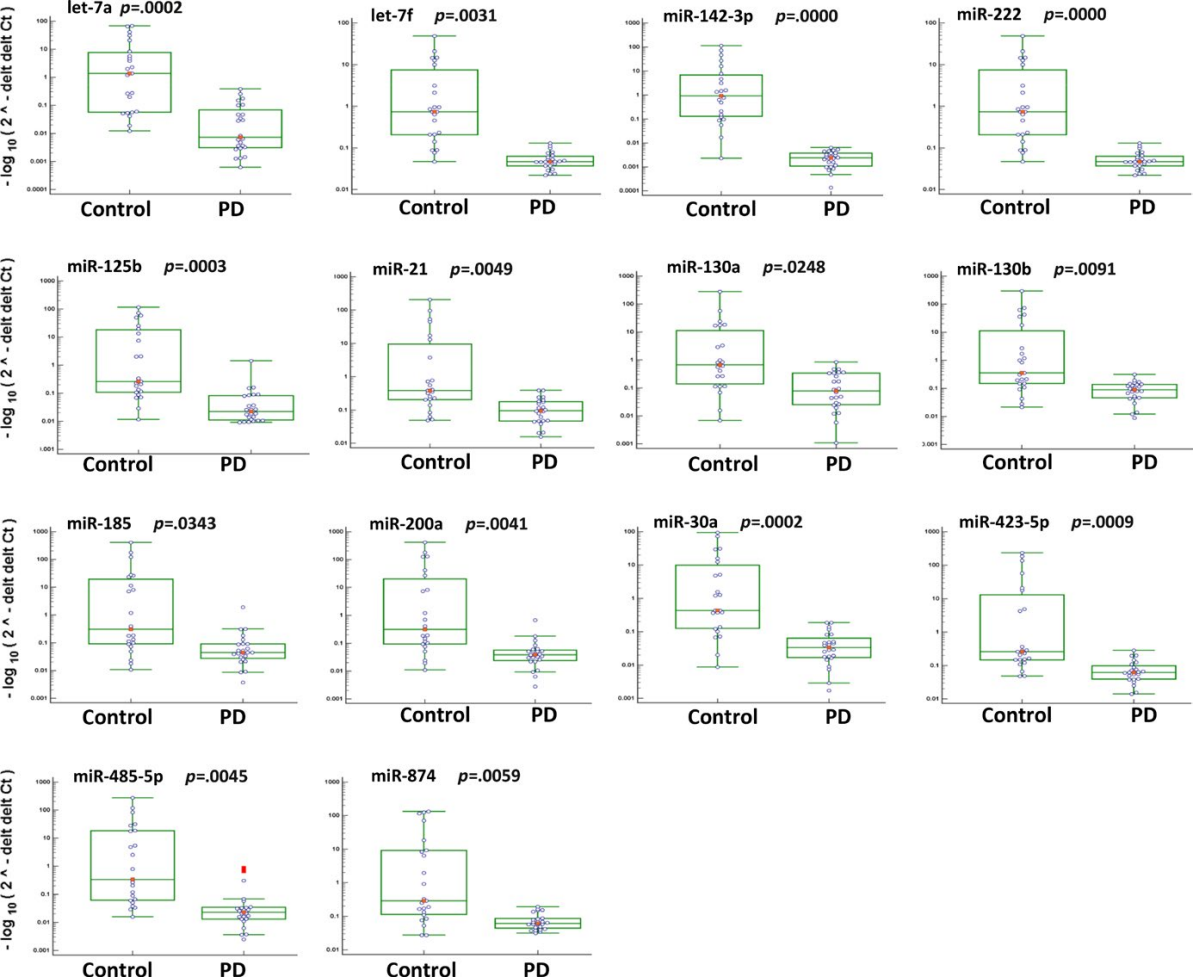
Scatter plot showing the expression levels of indicated miRNAs in all the tested samples. These miRNAs showed at least two folds less in the plasma of PD‐diagnosed patients than healthy controls, and adjusted *p* values ≤.05. The Mann–Whitney test was used for statistical analysis using IBM SPSS Statistics 21.0 software

Among the 91 tested miRNAs, 11 miRNAs including let‐7 g, miR‐1, miR‐10b, miR‐144, miR‐150, miR‐29a, miR‐34c, miR‐382, miR‐422a, miR‐433, and miR‐539 showed very low to undetectable levels in more than two‐third of the tested samples, which were excluded from further analysis. By application of fold change 2.0 cutoff and *p* value <.05, miR‐27a showed higher level (Figure 2), while 14 miRNAs showed lower levels (Figure 3) in the plasma samples of PD compared with normal controls. The 14 downregulated miRNAs include let‐7a, let‐7f, miR‐125b, miR‐130a, miR‐130b, miR‐142‐3p, miR‐185, miR‐200a, miR‐21, miR‐222, miR‐30a, miR‐423‐5p, miR‐485‐5p, and miR‐874.

**Figure 4:**
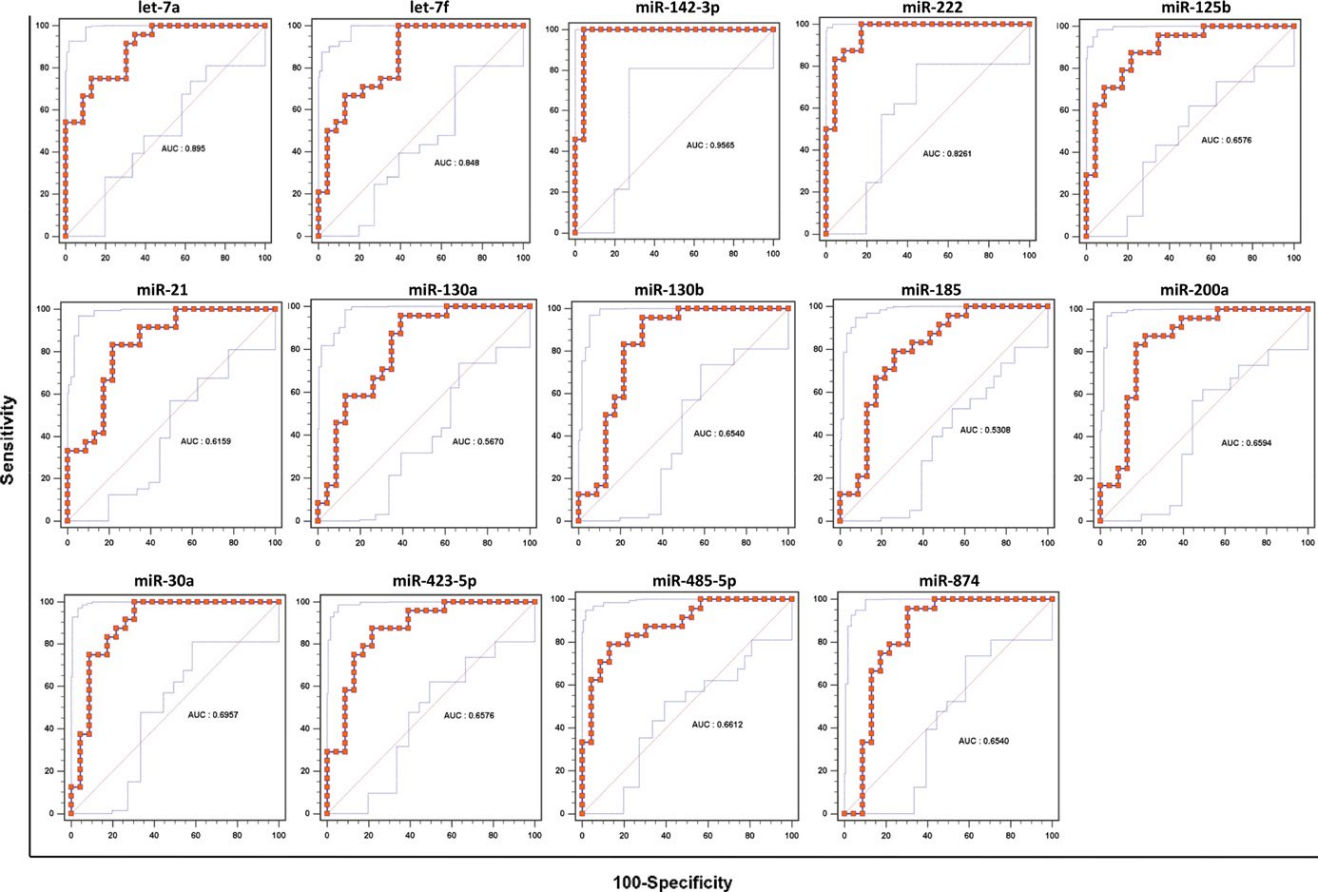
Receiver operating characteristic curves (ROC) of all 14 downregulated miRNAs, including let‐7a, let‐7f, miR‐142‐3p, and miR‐222 with the AUC values more than 0.8

In order to further determine the PD prediction capability of these miRNA, ROC curves were generated, and the AUC values were derived. As shown in Figure 2 and 4, upregulation of miR‐27a and downregulation of let‐7a, let‐7f, miR‐142‐3p, and miR‐222 in PD‐diagnosed patients showed AUC values more than 0.8, indicating high sensitivity and specificity to predict PD.

## 4 DISCUSSION

Circulating nucleic acids including DNA, mRNA, and miRNA can be detected in the plasma. They are released from blood cells, somatic cells, apoptotic cells, and/or exosomes. The circulating nucleic acids are mostly protected from degradation by binding with proteins or lipoprotein complexes. As such, circulating nucleic acids are highly stable in plasma and serum (Chen, Calin, & Meng, 2014; Korabecna, Pazourkova, Horinek, Rocinova, & Tesar, 2013). Although our understanding of the mechanism regulating the selective secretion of circulating RNAs remains unclear, circulating RNAs have been demonstrated to have potential roles in diagnosis and prognosis of various diseases (Chen, Lan, Roukos, & Cho, 2014; Cho, 2011; Korabecna et al., 2013; Turchinovich & Cho, 2014; Turchinovich, Tonevitsky, Cho, & Burwinkel, 2015). In addition, intake of circulating miRNAs by recipient cells has been demonstrated having function (Kosaka, Yoshioka, Hagiwara, Tominaga, & Ochiya, 2013).

Although the aberrant expression of miRNAs was reported frequently in PD‐diagnosed patients (Cardo et al., 2014; Filatova et al., 2012; Hoss et al., 2016), the circulating miRNA pattern in PD has not been well defined yet. Burgos et al. compared serum miRNA profiles in 67 PD‐diagnosed patients and 78 healthy controls, finding five deregulated miRNAs in PD‐diagnosed patients. The increased levels of miR‐338‐3p, miR‐30e‐3p, and miR‐30a‐3p and decreased levels of miR‐16‐2‐3p and miR‐1294 were detected (Burgos et al., 2014). In another study (Cardo et al., 2014), miR‐135b showed the most significant difference between patients and healthy donors. For the blood miRNAs in PD, Margis et al. (2011) reported that the expression levels of miR‐1, miR‐22*, and miR‐29 in blood allowed to distinguish nontreated PD from healthy subjects, while miR‐16, miR‐26a, and miR‐30a showed different levels in the blood samples between treated and untreated PD‐diagnosed patients. Herein, a customer‐designed miRNA panel containing 91 neuron disease‐related miRNAs was applied to compare the miRNA profiling in the plasma of PD‐diagnosed patients and healthy controls, identifying 15 deregulated circulating miRNAs in PD‐diagnosed patients. Among them, five miRNAs including miR‐27a, let‐7a, let‐7f, miR‐142‐3p, and miR‐222 showed high sensitivity and specificity to distinguish the PD‐diagnosed patients from the controls with good ROC curves and high AUC values.

Our results are consistent with the published studies. For example, decreased circulating miR‐30a level has been demonstrated in the untreated PD‐diagnosed patients compared to normal control and treated PD(Margis et al., 2011). Consistently, downregulation of miR‐30a in the plasma of PD‐diagnosed patients was identified in our current study (Figure 3). The different expression of miR‐30a was demonstrated in other central nerve system disorders including ischemic stroke, Huntington's disease, and schizophrenia (Darcq et al., 2015; Long et al., 2013). miRNA let‐7 family is highly conserved and is important and required for development, acting as regulator for oncogenes and stem cell differentiation (Bartel, 2009). Upregulation of let‐7 impairs glucose homeostasis and results in degeneration of neurons, while its downregulation leads to cancer (Shamsuzzama, Haque, & Nazir, 2016). let‐7 family members were underexpressed in the PD‐associated *Caenorhabditis elegans* models that either overexpress human A53T alpha‐synuclein or have mutations within parkin (pdr‐1) ortholog (Asikainen et al., 2010). Consistent with the literature, decreased levels of let‐7a and let‐7f in the plasma of PD were determined in our analysis. Furthermore, both let‐7a and let‐7f showed high sensitivity and specificity to detect PD with AUC values 0.895 and 0.848, respectively (Figure 4).

miR‐222 is associated with neurite outgrowth and immune function as a downstream target of the EGFR–RAS–RAF–MEK pathway. The overexpression of miR‐222 promotes premature cell cycle entry, leading to cell death. This could explain its possible role in the pathogenesis of neurodegenerative disorders, and in the apoptotic death of injured neurons (Teixeira, Gomes, & Medeiros, 2012). miR‐142‐3p has not been intensively studied in neurodegenerative diseases, but was found to be upregulated in the plasma samples of patients with Parkinson's disease (PD), and helped to distinguish Alzheimer's disease from PD (Cosín‐Tomás et al., 2017). This outcome is inconsistent with our own findings, but the authors of this previous study did not describe PD clinical characteristics such as stage and treatment. It is possible that plasma miR‐222 expression undergoes a dynamic change following disease progression and drug modification, which would explain the difference between the two studies. However, current knowledge is insufficient to explain how these miRNAs are involved in the pathogenic mechanisms of PD and their reciprocal association.

We are the first to identify miR‐27a as an upregulated circulating miRNA in the plasma of PD‐diagnosed patients (Figure 2). The identification of novel candidate of circulating miRNAs for PD diagnosis will be of great benefit to the patients getting appropriate treatment as early as possible. miR‐27a was suggested to represent potential therapeutic targets for PD (Kim et al., 2016). Loss‐of‐function mutations in PTEN‐induced putative kinase 1 (PINK1) and Parkin (PARK2) are the most common causes of autosomal recessive Parkinson's disease (PD). miR‐27 regulates the expression of PINK1 a protein associated with autophagic clearance of damaged mitochondria (Kim et al., 2016). Mitochondrial dysfunction is a key factor in several diseases including PD. Loss of PINK1 gene in mice significantly impairs mitochondrial function, which may lead to nigrostriatal degeneration in PD. The upregulation of miR‐27 might be one of the reasons leading to decreased expression of PINK1 which may mediate PD pathogenesis. It is yet to be determined whether the upregulation of circulating miR‐27a in PD is related to the race and/or location of the population.

It is feasible to detect miRNAs as biomarkers in the early stages of PD, but it has been challenging to use them for past clinical diagnoses. First, peripheral tissues excrete large amounts of different miRNAs, of which many are not organ‐specific. Second, miRNA concentrations become diluted when they cross from the cerebrospinal fluid through the blood–brain barrier. Finally, sample quality, measurement techniques, and data processing methods may affect the outcome accuracy. Parkinson's disease (PD) is thought to be a disease causing extensive damage to the central nervous system as well as the peripheral nervous system, and its pathogenesis is not exclusive to the brain (Braak, Rüb, Gai, & Del Tredici, 2003). With the development of modern sequencing and miRNA enrichment techniques, it is possible to differentiate miRNA levels between samples, even at very low concentrations (Pritchard, Cheng, & Tewari, 2012). For example, quantitative reverse transcription‐PCR can detect RNA levels as low as 10 ng, providing sensitive and accurate quantification (Aldridge & Hadfield, 2012). Advances in this field helped the development of more accurate and effective circulating miRNA tests.

In summary, our study provides a panel of novel miRNA candidates for detecting PD using plasma samples, further validation in larger independent cohorts is required before moving forward to clinical application.

## CONFLICTS OF INTEREST

The authors declare no conflict of interest.
