# Supplementary material for: Identification of aberrant circulating miRNAs in Parkinson's disease plasma samples
Source: Brain Behav. 2018 Feb 19;8(4):e00941. doi: 10.1002/brb3.941 (PMC5893342; doi:10.1002/brb3.941)

Supplemental Figure 1: Quality control of small RNA from plasma samples of normal control using Agilent 2100 bioanalyzer

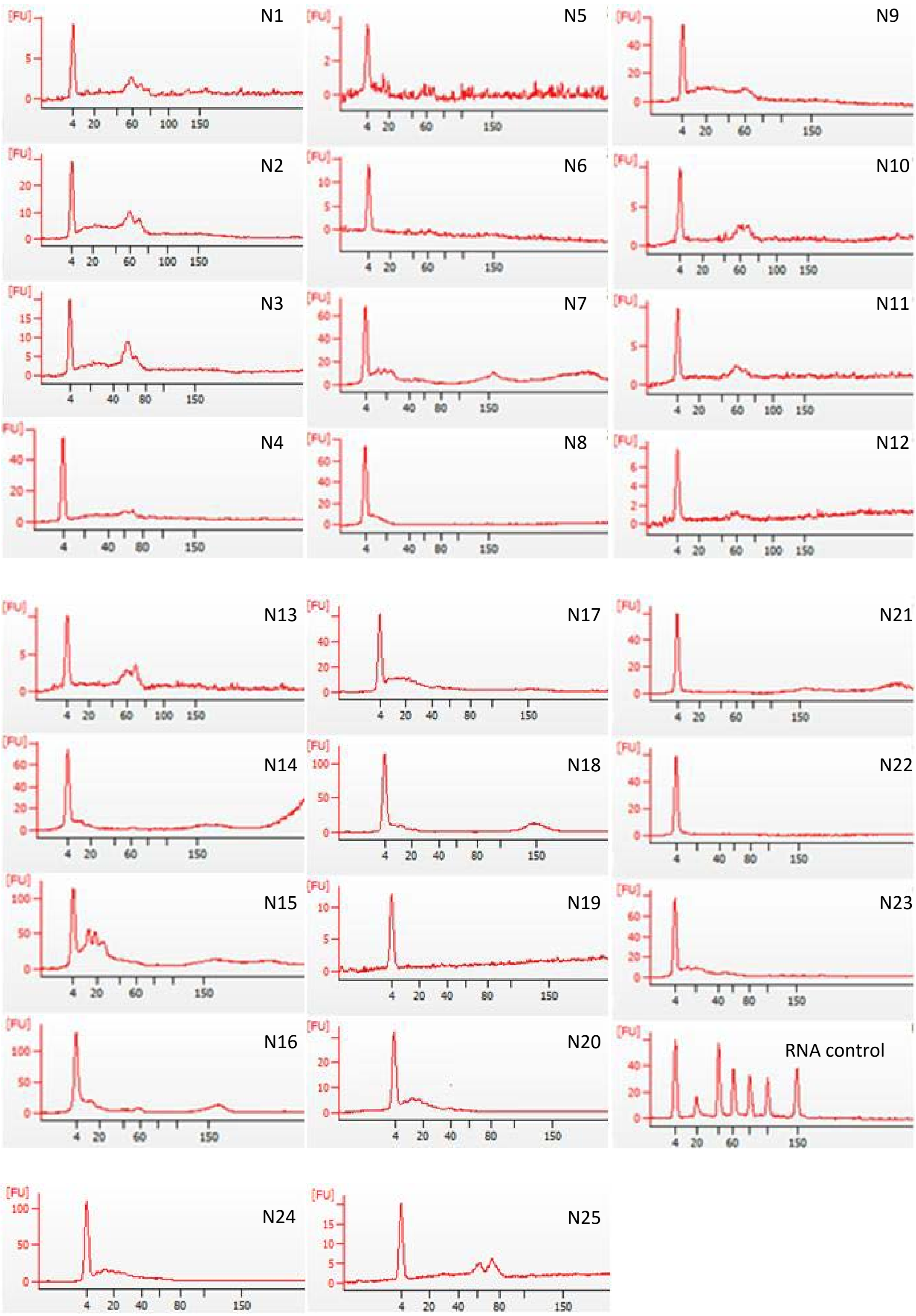

Supplemental Figure 2: Quality control of small RNA from plasma samples of PD patients using Agilent 2100 bioanalyzer

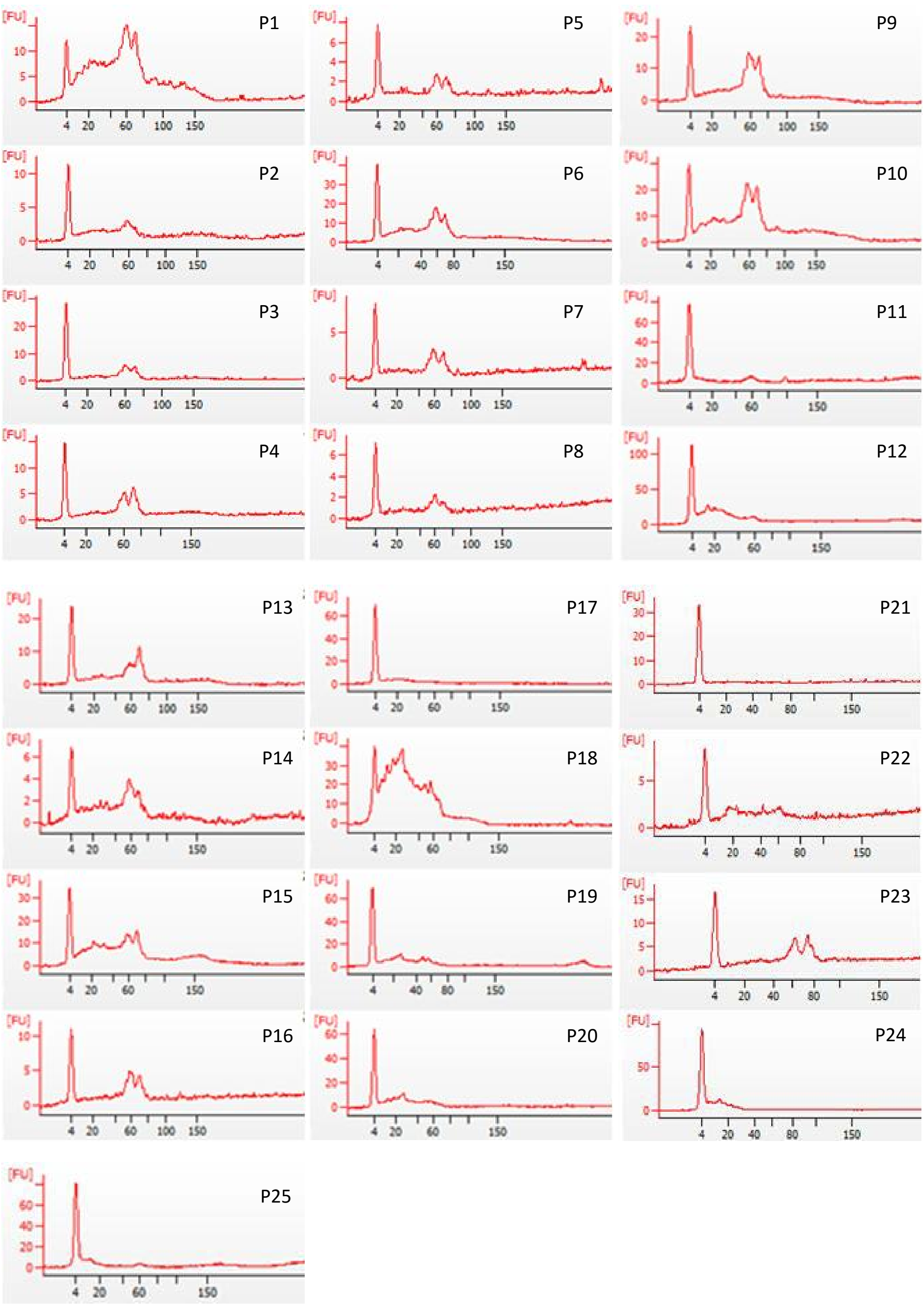

Supplement: Supplementary file 1 [file BRB3-8-e00941-s001.pdf]
